# Joint analysis of functionally related genes yields further candidates associated with Tetralogy of Fallot

**DOI:** 10.1038/s10038-022-01051-y

**Published:** 2022-06-20

**Authors:** Alexandru Chelu, Simon G. Williams, Bernard D. Keavney, David Talavera

**Affiliations:** Division of Cardiovascular Sciences, School of Medical Sciences, Faculty of Biology, Medicine and Health, The University of Manchester, Manchester, UK

**Keywords:** Congenital heart defects, Genetics research, High-throughput screening, Rare variants, Functional genomics

## Abstract

Although several genes involved in the development of Tetralogy of Fallot have been identified, no genetic diagnosis is available for the majority of patients. Low statistical power may have prevented the identification of further causative genes in gene-by-gene survey analyses. Thus, bigger samples and/or novel analytic approaches may be necessary. We studied if a joint analysis of groups of functionally related genes might be a useful alternative approach. Our reanalysis of whole-exome sequencing data identified 12 groups of genes that exceedingly contribute to the burden of Tetralogy of Fallot. Further analysis of those groups showed that genes with high-impact variants tend to interact with each other. Thus, our results strongly suggest that additional candidate genes may be found by studying the protein interaction network of known causative genes. Moreover, our results show that the joint analysis of functionally related genes can be a useful complementary approach to classical single-gene analyses.

Tetralogy of Fallot (TOF) is the most common cyanotic congenital heart defect (CHD) [1]. A full understanding of the aetiology of TOF has remained elusive, especially the major genetic mechanisms that contribute to the development of non-syndromic cases. Some genes have recently been identified as the main contributors to the development of TOF [2–6]. Nonetheless, those genes only explain a minority of cases. Many more genes are likely to be involved in the development of TOF, but novel approaches may be necessary to identify them. Causative genes are usually identified by comparing their allele frequencies in cases and controls, i.e., as a principle, causative variants should be found in cases but not in controls. These gene-by-gene survey studies encounter two main difficulties when applied to the study of oligogenic/polygenic diseases: (1) since many genes are involved in the disease, their individual effect size is pretty small for most of them, and (2) the total number of tests to perform creates a big multiple comparison burden. As a result, most single-gene tests lack statistical power [7, 8].

The joint analysis strategy attempts to overcome these difficulties: (1) the total number of tests is smaller, and (2) the effect size may be greater if the effects are in the same direction. Therefore, one essential consideration of a joint analysis is which genes need to be analysed together, and which ones should not be merged. Since most proteins interact with other proteins in order to carry out their function, we hypothesised that variants in either member of an interacting pair working together in a biological process might have similar effects. This hypothesis is supported by previous research that found that clusters of functionally related proteins were associated with particular diseases [9–11]. Indeed, Reuter et al. found that most genes known/suspected to be involved in TOF participate in a tightly packed protein interaction network [5]. In this study, we used the joint analysis approach in order to reanalyse whole-exome data from 829 patients of isolated, non-syndromic TOF. The cohort and the sequencing data have been described previously [4]. Instead of a gene-by-gene survey, we jointly analysed groups of genes that had been clustered based on current biological knowledge. This is an alternative approach to the network propagation method that has been used to identify new candidate genes interacting with genes known to be involved in a particular disease [12–14]. We grouped human proteins based on two conditions: (1) proteins had to participate in the same biological process as defined by the Gene Ontology [15, 16] (disregarding author/curator statements and electronic annotations), and (2) proteins had to physically interact with at least another protein within that group as reported by the BioGRID database [17, 18] (all reported interactions were included in the analysis). We focused our analysis on high-impact SNVs, i.e., single-nucleotide variants affecting splice sites, removing existent start or stop codons, or introducing novel stop codons. These variants are the most likely to affect the protein interaction network and hence the biological processes they participate in. Although moderate-effect variants (e.g., missense variants) may also be detrimental their effect on the biological processes is more difficult to assess. We used a permutation test in order to test if the number of patients with high-impact variants in particular groupings of functionally related genes was greater than those expected by chance in subsets of identical size (see Fig. 1 for a graphical representation of the analysis workflow). Similarly, we used a permutation test for assessing if there were more protein–protein interactions than expected by chance within the identified groupings of genes.

**Fig. 1:**
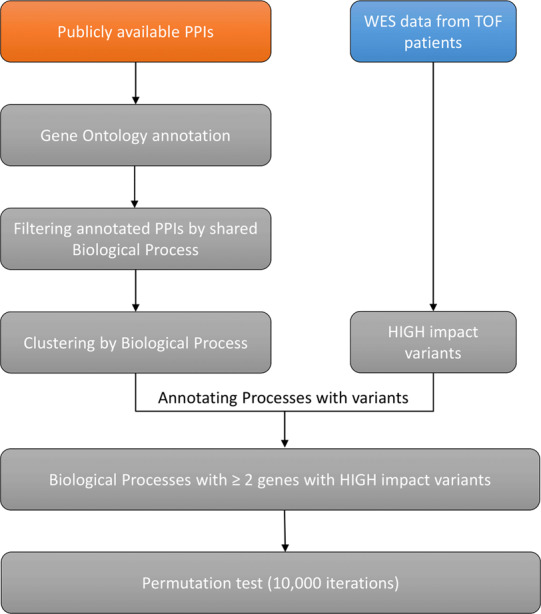
Diagram representing the analysis workflow. PPIs were extracted from the public repository. Each protein was annotated with the GO Biological Process terms assigned to them. Only PPIs where both proteins participate in the same biological process were kept. All biological process-based subnetworks of PPIs were identified. WES data from TOF patients were obtained. Only high-impact variants were kept for further analysis. PPIs subnetworks were annotated with the genetic variation information. Subnetworks containing at least two genes with high-impact variants were kept. A permutation test was used in order to assess if a particular subnetwork (biological process) was associated with more patients than expected by chance. The Python scripts used in this analysis are available from https://github.com/AlexUOM/JHG-Manuscript-code. PPIs protein–protein interactions, GO Gene Ontology, WES whole-exome sequencing, TOF Tetralogy of Fallot

Our results show that 12 functional groupings exceed the number of expected patients with high-impact variants (Bonferroni-adjusted *p* value < 0.01; Fig. 2A, B). Those 12 groupings contain 222 genes that were identified as having at least one high-impact variant in at least one patient. Although high-impact variants are likely to disrupt the protein function, we cannot be sure if the cell/organism can tolerate that effect. One way of assessing this is by studying if genes are under strong selective pressure, i.e., the number of variants observed in the population is smaller than expected [19]. Of those 222 genes, 69 have a pLI ≥0.9 (Supplementary Table I), showing enrichment for genes intolerant to loss-of-function (31.2% in the set of candidate genes vs 15.8% in the rest of the genome) (*p* value < 0.01; proportion test). A total of 165 patients (19.9 %) contain a high-impact variant in a single gene in those groupings, while 24 additional patients (2.9 %) contain variants in more than one gene. Thus, high-impact variants affecting those 12 functional groupings were found in 22.8% of the patients. In addition to biological processes assumed to be involved in TOF such as signalling pathways (19 patients) or regulation of transcription (130 patients), there were groupings involved in post-translational protein modification (41 patients), intracellular protein transport (10 patients), and cilium assembly (23 patients). This latter result in particular is in accord with the growing evidence showing that ciliopathies are linked to many cases of congenital heart disease, including TOF [3, 20]. Groupings are extremely sensitive to the functional annotations used, i.e., other annotation systems would likely lead to slightly different results (Supplementary Tables II and III and Supplementary Figs. S1–S3). Our results also confirmed our expectation that TOF-associated variants should be in interacting partners within the biological process (Fig. 2C, D and Supplementary Tables IV and V): interactions between proteins with high-impact variants exceed their expected-by-chance number in 8 out of the 12 groupings (*p* value < 0.05; Bonferroni-corrected permutation test), with some proteins bridging distinct biological processes.

**Fig. 2:**
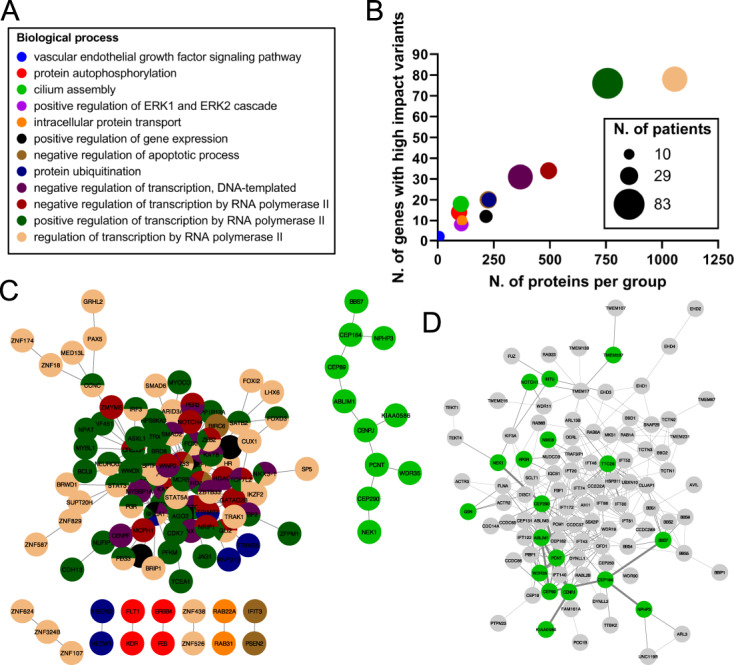
Functional groupings of genes associated with TOF. **A** List of functional groupings whose high-impact variants are associated with more patients than expected by chance. Colours are used to represent the functional groupings in the subsequent panels. **B** Description of the 12 statistically significant functional groupings. The scatter plot shows the number of proteins within each grouping, the number of genes with high-impact variants within the groupings and the number of patients with at least a variant in those groupings. **C** Network of protein interactions between proteins with high-impact variants. Out of the 222 proteins, 111 are present in the network. Nodes are coloured according to the within-biological process interactions. **D** Interaction network of the cilium assembly grouping. Green nodes represent proteins with high-impact variants, and grey nodes represent other proteins in the functional grouping. Interactions between proteins with high-impact variants are highlighted

Reassuringly, our approach recapitulated previous findings obtained in two gene-by-gene analyses of the same data [4, 5], i.e., we identified *FLT4*, *NOTCH1*, *KDR*, *JAG1* and *GATA6* as members of those functional groupings with high-impact variants. Indeed, 9 out of the 26 genes recently highlighted by Reuter et al. are members of these groupings. Importantly, we were also able to identify other genes involved in those functional groupings that had not previously been linked to TOF. Some of those genes had been associated with other types of CHD (e.g., *CEP290* [21], *KIAA0586* [21], *TCF12* [22]), or other cardiac phenotypes unrelated to TOF (e.g., *PSEN2* [23] and *CD36* [24]). Nonetheless, there was no known association between cardiovascular diseases and the vast majority of genes susceptible to altering the highlighted biological processes (e.g., *ARID3A, BRWD1, CBLC, CENPF*, *GSN, LHX6, MAP3K3, NRIP1, RNF213, TNIK, ZNF274*, *ZNF407*, and *ZNF808*).

Our analysis has not only been able to recapitulate the most prominent general biological processes known to be involved in the development of TOF such as signalling pathways and regulation of transcription but has also identified more specific processes known to be involved in CHD such as cilium assembly [3, 20]. Importantly, this approach can be used for highlighting possible candidates that would be overlooked in classical gene-by-gene analyses due to a lack of statistical power. Finally, our results suggest that the interaction partners of some known or emerging TOF candidates should be prioritised in future functional analyses. Our findings suggest that the joint analysis of groups of functionally related genes may be a powerful tool for identifying novel putative candidates involved in the development of congenital diseases.

## Supplementary information

Supplementary Table I

Supplementary Table II

Supplementary Table III

Supplementary Table IV

Supplementary Table V

Supplementary Figures S1–S3
