# Supplementary material for: Joint analysis of functionally related genes yields further candidates associated with Tetralogy of Fallot
Source: J Hum Genet. 2022 Jun 20;67(10):613–5. doi: 10.1038/s10038-022-01051-y (PMC7613636; doi:10.1038/s10038-022-01051-y)
Supplement: Supplementary file 2 — Supplementary Table II [file 10038_2022_1051_MOESM2_ESM.docx]

| **Biological process** | **Proteins with high impact variants** | **N. of proteins with high impact variants** | **N. of other proteins** | **Total proteins** | **Cases** | **CI** |
| --- | --- | --- | --- | --- | --- | --- |
| vascular endothelial growth  factor signaling pathway | FLT4, KDR | 2 | 4 | 6 | 11 | [0.0, 9.0] |
| protein autophosphorylation | STK11, RIPK3, VRK1, FES, MAP4K1, FLT4, INSRR, ERBB4, MAP3K3, MKNK1, FLT1, EPHB1, TNIK, KDR | 14 | 81 | 95 | 23 | [0.0, 18.327397959181326] |
| cilium assembly | CEP164, TTC26, RPGR, NEK1, INTU, BBS7, NME8, NOTCH1, GSN, TMEM237, NPHP3, CENPJ, WDR35, ABLIM1, CEP89, KIAA0586, PCNT, CEP290 | 18 | 85 | 103 | 23 | [0.0, 4.489846938775372] |
| positive regulation of  ERK1 and ERK2 cascade | NOTCH1, FLT4, DSTYK, PRKCZ, ERBB4,  CD36, PTPRC, KDR | 8 | 97 | 105 | 19 | [0.0, 17.817244897956698] |
| intracellular protein  transport | TBC1D17, RAB31, SNX27, STX11, VIPAS39, UNC93B1, USO1, RAB22A, TBC1D22A, SEC31A | 10 | 98 | 108 | 10 | [0.0, 9.0] |
| positive regulation of  gene expression | SRY, LEF1, FAM98B, NOTCH1, GSN, MYD88, BRCA1, CD36, STAT3, TP53, TTN, NKX3-1 | 15 | 200 | 215 | 15 | [0.0, 9.0] |
| negative regulation of  apoptotic process | IFIT3, LEF1, AREL1, SMAD6, BIRC6, FLT4, FXN, YME1L1, DSTYK, TJP1, PSEN2, TP53, NAA16, DNAJC3, THOC6, ANGPTL4, LRP2, DKK1, HDAC1, KDR | 20 | 205 | 225 | 26 | [0.0, 17.817244897956698] |
| protein ubiquitination | KCTD13, AREL1, OS9, CBLC, ZNF598, TRIM38, TRIM47, TRIM27, WWP2, RNF213, MYLIP, HERC5, FBXO4, BRCA1, TRIM10, KLHL8, HECW1, TRIM55, ASB1, HECW2 | 20 | 208 | 228 | 19 | [0.0, 17.817244897956698] |
| negative regulation of  transcription,  DNA-templated | TIMELESS, KAT8, HEXIM2, TCF7L2, ZNF224, HDAC10, ZNF174, KAT5, CENPF, NOTCH1, GATA6, WWP2, DPF1, CTBP2, 2 TP53, ELF3, FOXK2, YEATS2, RUNX1T1, HDAC9, SUV39H2, HDAC1, NKX3-1, LEF1, PER2, BRCA1, BCLAF1, ZBTB33, SMAD2, MYBBP1A, PHF10 | 31 | 337 | 368 | 54 | [0.0, 27.489846938775372] |
| negative regulation of  transcription by RNA polymerase II | SRY, MCPH1, HEXIM2, ZNF436, TCF7L2, GATAD2B, GLIS3, HDAC10, GLI2, RTF1, KAT5, NOTCH1, GATA6, AASS, WWP2, WWC2, TP53, DKK1, NRIP1, ZMYM5, YEATS2, FOXD3, NOTCH4, HDAC9, ZFPM1, SUV39H2, HDAC1, POU4F1, PARP9, PER2, TRIM27, BPTF, CREBBP, ZEB2 | 34 | 460 | 494 | 25 | [0.0, 18.327397959181326] |
| positive regulation of  transcription by RNA polymerase II | ZNF451, SRY, NUFIP1, CCNC, BRD8, GRHL2, KAT8, CAMTA2, HELZ2, AGO2, LPIN2, TCF7L2, GLIS3, MCRS1, SSBP2, BCL9, AGRN, ASXL1, GLI2, KAT5, IRF3, NOTCH1, NCOA1, CD40, GATA6, ATF6, DPF1, TCF12, MYOCD, CTBP2, 2 TP53, SLC9A1, ELF3, PGR, TET1, GABPB1, NEUROG1, JAG1, NRIP1, FOXK2, TFR2, ZNF407, SATB2, PEG3, CDK7, FOXD3, PFKM, MYBL1, STAT3, NPAT, ZFPM1, E2F8, TCEA1, ZNF335, HDAC1, POU4F1, RRP1B, NKX3-1, LEF1, RPS6KA3, ATMIN, NOS1, CDH13, CKAP2, ATRX, MLLT10, BPTF, CREBBP, ZEB2, BRCA1, BCLAF1, PRDM15, WWOX, SMAD2, PPP1R12A, PHF10 | 76 | 681 | 757 | 83 | [0.0, 9.0] |
| regulation of  transcription by RNA polymerase II | ARID3A, ZNF274, PAX5, CCNC, ZSCAN16, GRHL2, CAMTA2, SNF8, ZNF436, AHRR, TCF24, HR, TCF7L2, ZNF624, ZKSCAN5, GLI2, ZNF174, LIN9, IRF3, SMAD6, SRA1, KLF13, GATA6, ATF6, ZNF587, TCF12, TP53, ELF3, PGR, ZNF343, BATF2, NEUROG1, MED13L, FOXK2, ZNF829, STAT5A, BBS7, SATB2, PEG3, ZNF211, FOXD3, ZNF324B, HOXA3, FOXI2, ZNF226, STAT3, ZNF107, E2F8, LHX6, CUX1, POU4F1, HDAC1, BRIP1, NKX3-1, LEF1, EBF2, ATMIN, SUPT20H, ZNF213, ZNF18, TBX6, ISX, BPTF, ZEB2, BRWD1, ZSCAN5B, BRCA1, ZBTB33, ZNF808, SP5, ZNF438, PRDM15, TRAK1, ZBTB20, SMAD2, ZNF526, SIM1, IKZF2 | 78 | 978 | 1056 | 56 | [0.0, 17.817244897956698] |
