# Supplementary material for: Joint analysis of functionally related genes yields further candidates associated with Tetralogy of Fallot
Source: J Hum Genet. 2022 Jun 20;67(10):613–5. doi: 10.1038/s10038-022-01051-y (PMC7613636; doi:10.1038/s10038-022-01051-y)
Supplement: Supplementary file 3 — Supplementary Table III [file 10038_2022_1051_MOESM3_ESM.docx]

| **Biological process** | **Proteins with high impact variants** | **N. of proteins with high impact variants** | **N. of other proteins** | **Total proteins** | **Cases** | **CI** |
| --- | --- | --- | --- | --- | --- | --- |
| white fat cell differentiation | PER2, CTBP2 | 2 | 4 | 6 | 28 | 0.0, 27.0 |
| cellular response to vascular endothelial growth factor stimulus | FLT4, NOTCH1, KDR, FLT1 | 4 | 16 | 20 | 15 | 0.0, 9.57987394958036 |
| regulation of cell migration | PLXNA1, AMOTL1, LMNA, PLXNB2, KIF2A, PLXND1, ERBB4 | 7 | 29 | 36 | 7 | 0.0, 5.0 |
| transmembrane transport | ABCC8, SLC4A7, ANO4, SLC4A10, ABCB8, ABCC5, ANO7, ANO3, SLC4A2 | 9 | 29 | 38 | 10 | 0.0, 9.0 |
| positive regulation of endothelial cell proliferation | FLT4, CDH13, KDR, EGR3, STAT5A | 5 | 33 | 38 | 14 | 0.0, 9.0 |
| positive regulation of kinase activity | FLT4, KDR, EPHB4, EPHB2, RYK, HLA-DRB1, DSTYK, INSRR, MUSK, FLT1, EPHB1, MERTK, ERBB4 | 13 | 47 | 60 | 21 | 0.0, 8.1597478991607 |
| adenylate cyclase-activating G protein-coupled receptor signaling pathway | TSHR, GPR4, GHRHR, ABCA1, GCGR | 5 | 76 | 81 | 6 | 0.0, 5.0 |
| actin filament organization | BCAR1, MYO15A, MYO7B, TPM1, MARCKSL1, MYO7A, MYO1C, CLRN1, ARHGAP17 | 9 | 72 | 81 | 12 | 0.0, 5.0 |
| axon guidance | PTPRO, SEMA5B, ARHGAP35, EPHB4, EPHB2, ROBO3, ROBO2, SEMA6C, EFNB2, NOTCH1, RYK, ENAH, CNTN4, NOTCH3, EPHB1, UNC5A, IGSF9, SLIT3, SEMA4D, DSCAML1, SEMA6B, GLI2 | 22 | 67 | 89 | 27 | 0.0, 10.73962184874108 |
| positive regulation of GTPase activity | CD40, TBC1D2, NF1, USP6NL, ARAP1, DOCK8, NDEL1, SEMA4D, DOCK9, AGRN | 10 | 86 | 96 | 8 | 0.0, 7.319495798321441 |
| proteolysis | CAPN8, ERAP2, XPNPEP1, TMPRSS2, MME, PRSS22, CAPN1, HGFAC, YME1L1, CTSC, F12 | 11 | 105 | 116 | 12 | 0.0, 9.0 |
| protein autophosphorylation | EPHB4, MUSK, FLT1, FLT4, TNIK, INSRR, CDK12, MAP4K1, NEK6, MKNK1, WNK2, EPHB1, RIPK3, FES, VRK1, ERBB4, STK33, KDR, STK11, MAP3K3, MYO3A | 21 | 109 | 130 | 29 | 0.0, 27.0 |
| positive regulation of cell migration | BCAR1, SEMA5B, LEF1, APC, FLT1, AIF1, STAT3, SEMA6C, NOTCH1, COL1A1, MYO1C, LAMB1, CDH13, KDR, SEMA4D, SEMA6B, SUN2, SMO, TJP1 | 19 | 135 | 154 | 19 | 0.0, 9.0 |
| cell adhesion | PCDHB3, EPHB4, PCDH20, COL16A1, PCDHA6, VWF, PCDHGB7, ITGA9, POSTN, EFNB2, CDHR5, PCDHGC3, IGFBP7, PCDHGA9, ICAM5, FES, VCAM1, PCDHA5, PCDHGA10, IBSP | 20 | 136 | 156 | 16 | 0.0, 9.57987394958036 |
| intracellular signal transduction | STK32C, NUAK1, JAK3, WNK1, NRG1, PRKCZ, DGKB, TNIK, SMAD2, NEK11, MKNK1, IRAK2, WNK2, STK38L, SH2B3, MAP3K3 | 16 | 140 | 156 | 19 | 0.0, 9.57987394958036 |
| protein phosphorylation | NUAK1, HCST, CDK7, WNK1, CDC25B, CDK20, PRKCZ, TNIK, MAP3K6, MAP4K5, ZAP70, CDK12, MAP4K1, NEK6, NEK11, MKNK1, NEK1, RYK, WNK2, VRK1, PRKACB, STK38L, STK11 | 23 | 239 | 262 | 23 | 0.0, 19.437731092446484 |
| negative regulation of apoptotic process | GATA6, THOC6, SMAD6, NAT8, IFIT3, LRP2, ANGPTL4, LEF1, FLT4, FXN, BIRC6, DKK1, YME1L1, TP53, DSTYK, NAA16, PSEN2, DNAJC3, HDAC1, KDR, AREL1, FSTL1, TJP1 | 23 | 240 | 263 | 29 | 0.0, 9.0 |
| positive regulation of cell population proliferation | NRG1, NKX3-1, TBX6, LEF1, REG1A, FLT4, CRLF1, AIF1, EFNB2, GHRHR, TET1, NOTCH1, TRPM4, UFL1, ERBB4, HDAC1, KDR, FAM98B, GAB2, TJP1 | 20 | 269 | 289 | 29 | 0.0, 7.319495798321441 |
| positive regulation of gene expression | EPHB2, NAT8, CD36, NKX3-1, LEF1, TTN, MUSK, AMH, DKK1, STAT3, TP53, POU4F1, NOTCH1, GCGR, BRCA1, GSN, SNF8, FAM98B, MYD88, SMO | 20 | 276 | 296 | 21 | 0.0, 9.0 |
| negative regulation of transcription, DNA-templated | TP53, SMAD2, CENPF, BCLAF1, KAT8, TIMELESS, DPF1, KAT5, SUMO4, ZNF541, TCF7L2, HEXIM2, SETDB2, ELF3, PER2, LOXL2, RUNX1T1, HDAC9, HDAC10, MYBBP1A, ZBTB33, GATA6, NRG1, NKX3-1, LEF1, CTBP2, 2ACTRT1, SUV39H2, ZNF224, NOTCH1, FOXK2, WWP2, BRCA1, HDAC1, ZNF174, YEATS2, PHF10 | 37 | 381 | 418 | 59 | 0.0, 5.57987394958036 |
| positive regulation of transcription, DNA-templated | CREBBP, PARP9, BRPF1, TP53, SMAD2, KAT8, NAA16, KAT5, TRIP4, HLA-DRB1, STAT3, MSTN, ERBB4, ATMIN, NCOA1, COL1A1, GATA6, NKX3-1, LEF1, MYOCD, CEP290, NOS1, NOTCH1, FOXK2, BRCA1, HDAC1, NPAT, GLI2, RGMB | 29 | 426 | 455 | 24 | 0.0, 19.437731092446484 |
| negative regulation of transcription by RNA polymerase II | WWC2, CREBBP, PARP9, ZMYM5, TP53, SEMA4D, KAT5, TRIM27, DKK1, POU4F1, TCF7L2, AASS, NRIP1, HEXIM2, FOXD3, ZEB2, PER2, ZFPM1, LOXL2, HDAC9, TBX6, RTF1, HDAC10, PEG3, BPTF, MCPH1, GLIS3, SKOR1, GATA6, NOTCH4, SUV39H2, ZNF224, SKOR2, NOTCH1, WWP2, GATAD2B, HDAC1, ZNF174, YEATS2, E2F8, GLI2 | 41 | 501 | 542 | 29 | 0.0, 19.437731092446484 |
| positive regulation of transcription by RNA polymerase II | CD40, CREBBP, RRP1B, ATRX, PFKM, PPP1R12A, TCEA1, TP53, PRDM15, SMAD2, RPS6KA3, BCLAF1, ZNF407, KAT8, SLC9A1, CSRNP3, BRD8, DPF1, KAT5, FSTL3, SATB2, ATF6, STAT3, POU4F1, TCF7L2, NRIP1, HELZ2, FOXD3, ELF3, ZEB2, ZFPM1, MCRS1, ATMIN, CDK7, PEG3, BCL9, MYBL1, ZNF451, AGO2, TET1, BPTF, NCOA1, SSBP2, GABPB1, CCNC, JAG1, GLIS3, AGRN, ZNF335, GATA6, ASXL1, NKX3-1, LEF1, TCF12, CTBP2, 2GRHL2, MLLT10, WWOX, PGR, MYOCD, TFR2, NOS1, FOXK2, NOTCH1, LPIN2, NEUROG1, IRF3, BRCA1, HDAC1, CDH13, CAMTA2, E2F8, NPAT, PHF10, GLI2, NUFIP1 | 76 | 730 | 806 | 85 | 0.0, 27.0 |
| regulation of transcription by RNA polymerase II | ZNF211, DRGX, CC2D1B, TP53, HOXA3, SMAD2, ISX, ZKSCAN5, ARID3A, ZNF526, ZNF829, LHX6, CSRNP3, MYNN, ZSCAN16, ZNF208, SATB2, ATF6, SIM1, STAT3, ZNF541, POU4F1, TCF7L2, ZBTB20, BATF2, ZSCAN5B, HR, EBF2, SP5, AHRR, FOXD3, KLF13, HMGB4, ELF3, ZEB2, MED13L, EGR3, TBX6, TCF24, CUX1, PAX4, SUPT20H, ZNF236, PEG3, ZNF587, ZNF18, STAT5A, ZNF324B, ZNF850, ZNF551, BPTF, ZNF808, LIN9, ZSCAN2, SNF8, CCNC, GLIS3, ZBTB33, BRWD1, GATA6, ZNF624, SMAD6, NKX3-1, ZNF213, LEF1, TCF12, GRHL2, IKZF2, ZNF382, BBS7, PAX5, ZNF274, PGR, RFX8, ZNF224, FOXK2, ZNF107, NEUROG1, IRF3, BRCA1, HDAC1, ZNF99, BRIP1, CAMTA2, ZNF174, FOXI2, E2F8, GLI2, TRAK1, SRA1 | 90 | 1089 | 1179 | 71 | 0.0, 27.57987394958036 |
