# Supplementary material for: Joint analysis of functionally related genes yields further candidates associated with Tetralogy of Fallot
Source: J Hum Genet. 2022 Jun 20;67(10):613–5. doi: 10.1038/s10038-022-01051-y (PMC7613636; doi:10.1038/s10038-022-01051-y)
Supplement: Supplementary file 6 — Supplementary Figures S1–S3 [file 10038_2022_1051_MOESM6_ESM.docx]

**Supplementary Figures S1-S3**

**Figure S1. Effect of data sources on the composition of groupings.** Rows refer to significant groupings identified using BioGRID as the source for PPIs. Columns refer to significant groupings identified using STRING as the source for PPIs. Groupings were compared using the Jaccard distance; i.e. 1 – Jaccard Index. Identical sets have a distance of 0, while non-overlapping sets have a distance equal to 1. Groupings were compared and clustered within experiment (i.e. with the same database), and between experiments. Dendrograms show the similarity of within-experiment groupings, while the heatmap shows the similarity of between-experiments groupings.

**Figure S2. Effect of data sources on the identification of candidate genes.** Identical to Figure S1, but only genes with HIGH impact variants were included in the analysis. Rows refer to significant groupings identified using BioGRID as the source for PPIs. Columns refer to significant groupings identified using STRING as the source for PPIs. Groupings were compared using the Jaccard distance; i.e. 1 – Jaccard Index. Identical sets have a distance of 0, while non-overlapping sets have a distance equal to 1. Groupings were compared and clustered within experiment (i.e. with the same database), and between experiments. Dendrograms show the similarity of within-experiment groupings, while the heatmap shows the similarity of between-experiments groupings.

**Figure S3. Effect of data sources on the individual subnetworks.** A. Cillium assembly subnetworks built using BioGRID data (110 proteins – 328 interactions – 3 subnetworks). B. Cillium assembly subnetworks built using STRING data (137 proteins – 679 interactions – 2 subnetworks). C. Axon guidance subnetworks built using BioGRID data (43 proteins – 43 interactions – 9 subnetworks). D. Axon guidance subnetworks built using STRING data (91 proteins – 496 interactions – 2 subnetworks). Each subnetwork was analysed individually. STRING data resulted in bigger tighter subnetworks. Differences in the results obtained using BioGRID or STRING are likely due to those extra proteins. If the STRING network contains additional proteins with HIGH impact variants this will increase the likelihood of a significant result. Alternatively, if the extra proteins do not contain HIGH impact variants, the likelihood will decrease.
